# *Senna singueana*: Antioxidant, Hepatoprotective, Antiapoptotic Properties and Phytochemical Profiling of a Methanol Bark Extract

**DOI:** 10.3390/molecules22091502

**Published:** 2017-09-08

**Authors:** Mansour Sobeh, Mona F. Mahmoud, Rehab A. Hasan, Haroan Cheng, Assem M. El-Shazly, Michael Wink

**Affiliations:** 1 Institute of Pharmacy and Molecular Biotechnology, Heidelberg University, Im Neuenheimer Feld 364, Heidelberg 69120, Germany; haorancheng2007@gmail.com; 2 Department of Pharmacology and Toxicology, Faculty of Pharmacy, Zagazig University, Zagazig 44519, Egypt; mona_pharmacology@yahoo.com; 3 Department of Histology, Faculty of Medicine for Girls, Al-Azhar University, Cairo 11651, Egypt; rehababduallah@yahoo.com; 4 Department of Pharmacognosy, Faculty of Pharmacy, Zagazig University, Zagazig 44519, Egypt; assemels2002@yahoo.co.uk

**Keywords:** *Senna singueana*, proanthocyanidins, HPLC-PDA-ESI-MS/MS, antioxidant, hepatoprotection, *Caenorhabditis elegans*

## Abstract

Natural products are considered as an important source for the discovery of new drugs to treat aging-related degenerative diseases and liver injury. The present study profiled the chemical constituents of a methanol extract from *Senna singueana* bark using HPLC-PDA-ESI-MS/MS and 36 secondary metabolites were identified. Proanthocyanidins dominated the extract. Monomers, dimers, trimers of (epi)catechin, (epi)gallocatechin, (epi)guibourtinidol, (ent)cassiaflavan, and (epi)afzelechin represented the major constituents. The extract demonstrated notable antioxidant activities in vitro: In DPPH (EC_50_ of 20.8 µg/mL), FRAP (18.16 mM FeSO_4_/mg extract) assays, and total phenolic content amounted 474 mg gallic acid equivalent (GAE)/g extract determined with the Folin-Ciocalteu method. Also, in an in vivo model, the extract increased the survival rate of *Caenorhabditis elegans* worms pretreated with the pro-oxidant juglone from 43 to 64%, decreased intracellular ROS inside the wild-type nematodes by 47.90%, and induced nuclear translocation of the transcription factor DAF-16 in the transgenic strain TJ356. Additionally, the extract showed a remarkable hepatoprotective activity against d-galactosamine (d-GalN) induced hepatic injury in rats. It significantly reduced elevated AST (aspartate aminotransferase), and total bilirubin. Moreover, the extract induced a strong cytoplasmic Bcl-2 expression indicating suppression of apoptosis. In conclusion, the bark extract of *S. sengueana* represents an interesting candidate for further research in antioxidants and liver protection.

## 1. Introduction

Reactive oxygen species (RO), which include hydroxyl radical, hydrogen peroxide, nitric oxide, and superoxide, are highly reactive molecules which originate as a by-product of normal cell metabolism from the uncoupled mitochondrial electron transport chain, from various cellular enzymes including xanthine oxidase and NADPH oxidases as well as from external sources such as exposure to radiation, organic compounds, cigarette smoking, and air pollutants [1]. ROS can be mutagenic by converting the nucleotide base guanosineinto 8-oxoguanosine. Overexposure of the body to ROS apparently plays a major part in the progression of aging, aging-related degenerative diseases including inflammation, diabetes, hepatitis, cardio-vascular conditions, and cancer [2,3].

The body has a natural defense against free radicals such as enzymatic antioxidants including superoxide dismutase and catalase as well as non-enzymatic antioxidants such as glutathione, vitamin C and E. Natural phytochemicals, among them polyphenols and carotenoids, also play important roles in ROS scavenging due to their ability to act as hydrogen donors, and reducing agents. Hence, nutraceuticals with antioxidant properties have received considerable interest because of their potential to modulate oxidative stress and ROS related health conditions [4,5,6].

The genus *Senna*, with about 600 species, is one of the largest genera belonging to the Fabaceae and was formerly included in the genus *Cassia*. It comprises shrubs and trees and is widely spread in the tropics [7]. Several classes of plant secondary metabolites have been reported in this genus including flavonoids, anthraquinones, stilbenes, terpenes, alkaloids, and proanthocyanidins [8].

*Senna singueana* Delile (syn. *Cassia singueana*), commonly known as wild *Cassia*, is a traditional African medicinal plant. Throughout Africa, the plant is used traditionally to treat diabetes, stomach pains, leprosy, rheumatism, microbial and sexual transmitted infections, and inflammation as well as fever, skin cancer, and malaria [9,10]. In the rural area of the south-eastern Sudan, a plant root decoction is used to treat constipation [11]. The leaves and bark extracts from northern Ethiopia species were shown to scavenge free radicals and to inhibit erythrocyte hemolysis [12].

In Nigeria, several studies reported the antioxidant activities and furthermore, anti-diabetic activities of the acetone fraction of stembark and these activities were attributed to the presence of resorcinol and dibenzofuran [13,14]. Also, the root bark showed promising antimalarial activity against *Plasmodium berghei* as well as antipyretic and antinociceptive activities, and the authors related these activities to the presence of tannins, saponins and some traces of anthraquinones [15].

Furthermore, the leaves from Northern Ethiopia showed promising antimalarial activities alone or in a combination with chloroquine [16]. The root bark from plants from Kenya showed antinociceptive activities in vivo and the leaves from plants growing in southern Malawi were safe when investigated using brine shrimp lethality [17,18]. The bark extract from Tanzania showed trypanocidal activities [19]. Preliminary phytochemical analysis of the leaves, seed and root revealed several classes of plant secondary metabolites including phenols, saponins, tannins, and anthraquinones as well as alkaloids, tannins, sterols, and terpenes [20,21,22].

The current work comprehensively profiled the chemical constituents of the methanol extract using HPLC-PDA-ESI-MS/MS. The antioxidant activity of the extract was investigated in vitro and in vivo using the model system *C. elegans*, which is widely used in this context [23]. In addition, the hepatoprotective activities of the bark extract in d-galactosamine-induced liver toxicity in rats was evaluated.

## 2. Results and Discussion

### 2.1. Secondary Metabolites in the Methanol Extract from S. singueana Bark

**Figure 1:**
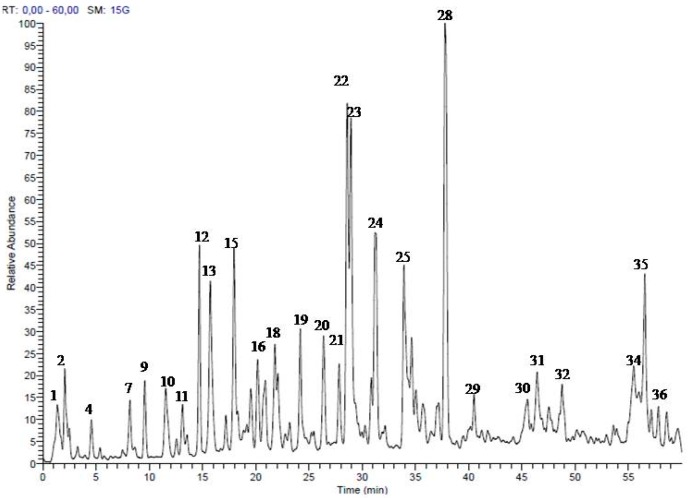
LC-MS profile of the methanol extract of *S. singueana* bark (LC-MS base peak in the negative ion mode).

HPLC-PDA-ESI-MS/MS is a powerful analytical technique for profiling the polyphenolics in complex extracts [24]. Analyses of the methanol bark extract in a negative ion mode allowed the identification of 36 compounds belonging mainly corresponding to flavanols differing in the degree of polymerization and isomerization. Oligomers (dimers and trimers) of (epi)gallocatechin, (epi)catechin, (epi)guibourtinidol, (ent)cassiaflavan, and (epi)afzelechin represented the major compounds in the extract. For instance, proanthocyanidins dimers consists of (epi)gallocatechin-(epi)catechin, (epi)catechin-(epi)catechin, (epi)catechin-(epi)afzelechin, and (epi)guibourtinidol-(epi)catechin; they were identified according to typical MS data, such as ions at [M − H]^−^ *m*/*z* 593, 577, 561, and 545, respectively [25,26,27,28]. MS data and retention times are presented in Figure 1 and Table 1.

**Figure 2:**
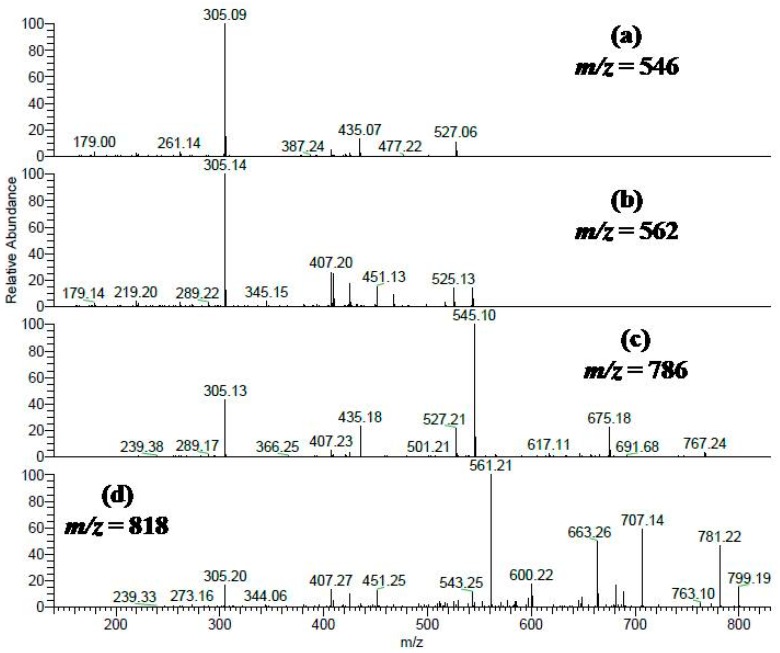
MS^2^fragmentation pattern of selected polyphenols from compounds listed in Table 1. (**a**) MS/MS spectrum of deprotonated compounds **22** and **23** at *m*/*z* 545; (**b**) MS/MS spectrum of deprotonated compounds **13**, **15**, **16**, and **18** at *m/z* 561; (**c**) MS/MS spectrum of deprotonated compounds **31**, **34**, and **35** at *m/z* 785; (**d**) MS/MS spectrum of deprotonated compound **26** at *m*/*z* 817.

Two peaks (**22** and **23**) showed [M − H]^−^ at *m*/*z* 545 and a main fragment ion at 305 (M − H −240); they were identified as (ent)cassiaflavan-(epi)gallocatechin as previously described [28]. A representative mass spectrum is shown in Figure 2a (*m*/*z* 546). Furthermore, four peaks (**13**, **15**, **16**, and **18**) gave a molecular ion peak at *m*/*z* 561 and a main fragment ion at305 (M − H −256); they were tentatively assigned to (epi)guibourtinidol-(epi)gallocatechin, a spectrum is shown in Figure 2b (*m*/*z* 562). Additionally, compound **28** showed a [M − H]^−^ at *m*/*z* 529 and a main fragment ion at 289 (M − H −240), it was assigned to (ent)cassiaflavan-(epi)catechin [28]. Another compound **36** was tentatively identified as (ent)cassiaflavan-(ent)cassiaflavan-(epi)catechin based on its [M − H]^−^ *m*/*z* 769 and fragment ions 529 (M − H −240, loss of (ent)cassiaflavan moiety), and 289 (M − H −240 −240, loss of two (ent)cassiaflavan moieties).

Figure 2c shows a representative mass spectrum of a molecular ion peak at [M − H]^−^ *m*/*z* 785 (compounds **31**, **34**, and **35**) with two consecutive losses of 240 Da, yielding fragment ions at *m*/*z* 545 and 305; they were tentatively assigned to (ent)cassiaflavan-(ent)cassiaflavan-(epi)gallocatechin. In peak **26** (Figure 2d; *m*/*z* 818), a molecular ion peak was detected at [M − H]^−^ *m*/*z* 817 and in MS^2^ showed fragments ions at 561 (M − H −256) and 305 (M − H −256 −256); the compound was tentatively identified as (epi)guibourtinidol-(epi)guibourtinidol-(epi)gallocatechin. In addition, we detected monomers of (epi) catechin, (epi)gallocatechin, and organic acids [25,26].

### 2.2. Biological Activities

#### 2.2.1. Antioxidant Activities

We determined the antioxidant activities of the methanol extract in vitro using DPPH and FRAP assays. Substantial antioxidant activities were detected in both assays and represented by an EC_50_ of 20.8 µg/mL in DPPH compared to the positive control epigallocatechin gallate (EGCG) (EC_50_ of 3.50 µg/mL) and 18.16 mM FeSO_4_/mg extract in FRAP with regard to 25 mM FeSO_4_ of the reference compound EGCG. These solid antioxidant activities might be attributed to its high total phenolic content as it found to be 474 mg gallic acid equivalent (GAE)/gm extract when estimated by the Folin-Ciocalteu method. Similar results were detected in extracts from *Cassia abbreviata* and other *Senna* species rich in proanthocyanidins [29,30].

**Figure 3:**
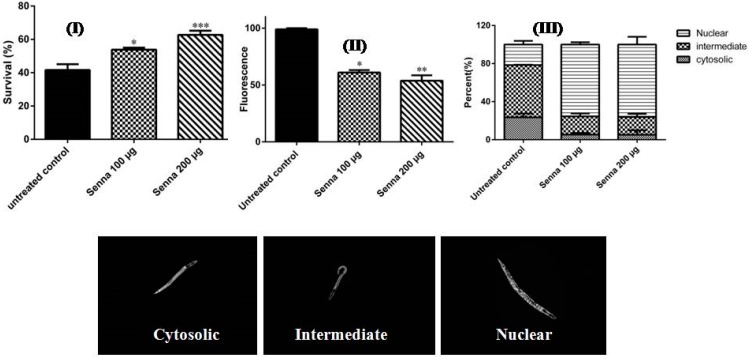
(**I**) Protective effects in N2 worms pre-treated with *S. singueana* on the survival rate of wild-type nematodes treated with a lethal dose of 80 μM juglone. Significant increase in the survival rate was observed after the worms were pre-treated with the extract. Data are presented as percentage of survival (mean ± SEM, *n*=3) (**II**): Intracellular ROS levels in wild-type N2 nematodes evidenced by the fluorescence H2DCF-DA dye. Results are presented as relative fluorescent intensity related to control of the mean ± SEM of three individual experiments in which at least 20 worms were analyzed. The accumulation of mitochondrial ROS was significantly diminished when worms were pre-treated with the extract (**III**): Effects of the root extract on the translocation of DAF-16 transcription factor in mutant TJ356 strains. Subcellular localization of DAF-16:GFP was analyzed by fluorescence microscopy (200×) and the nematodes were sorted to the categories cytosolic, intermediate, and nuclear according to their localisation phenotype as shown in the representative images.* *p* < 0.05, ** *p* < 0.01,*** *p* < 0.001 related to control was analysed by one-way ANOVA.

To monitor whether these in vitro antioxidant capacities come into effect in vivo, the antioxidant properties were studied in the model organism *C. elegans*, a suitable model for pharmacological activities [31]. The lyophilized extract (100 µg/mL and 200 µg/mL) showed an increase in the survival rate of the worms treated with the pro-oxidant juglone (N2 type) from 43.54 to 57.17% and from 43.54 to 64.29%, respectively. To assess whether the antioxidant properties of the studied extract is reflected into reduced ROS production, the level of intracellular ROS was measured in wild-type nematodes against elevated ROS levels induced by juglone. The extract was able to scavenge the reactive oxygen species, evidenced by a decrease in intracellular ROS in which was attenuated in a dose dependent manner with 36.76% and 47.90% by 100 and 200 µg/mL, respectively, compared to the untreated control group (untreated control refers to worms treated only with juglone). Results are illustrated in Figure 3 (I and II). Similar pattern was found in several crude plant extracts and other phenolic compounds [32,33].

In *C. elegans*, plant extracts or compounds which are able to activate the FOXO transcription factor (DAF-16) increase the life span [34]. When activated, the transcription factor translocates from the cytosol into the nucleus to induce stress related genes [31]. The studied extract (100 and 200 µg/mL) was able to induce nuclear translocation in the transgenic strain TJ356 up to 76.19% compared with the untreated worms (21.56%). Representative pictures of the localization of DAF-16 (nuclear, intermediate and cytosolic) are illustrated in Figure 3III. Similar mechanisms have been reported for other plant polyphenolics and proanthocyanidin rich extracts [23,33,34,35].

#### 2.2.2. Hepatoprotective Activity

Two types of liver conditions are distinguished, acute and chronic liver diseases. In most cases, acute liver disease (ALF) resolves spontaneously but it may develop into some other health disorders. ALF is a severe and fatal condition, which is characterized by a rapid deterioration of liver function. It is also associated with extensive morbidity and increased rate of mortality. ALF is associated with the death of most hepatocytes [36]. However, the liver is characterized by a high regenerative capacity following injury. Hepatocyte death can occur via various processes, including apoptosis, necrosis or autophagy. When liver is exposed to viruses, drugs, alcohol, toxins and ischemic injury, programmed cell death or necrosis are often observed [37].

d-GalN is a hepatotoxic agent, inducing severe hepatic injury. d-GalN treatment mimics viral hepatitis which leads to liver failure and hepatic carcinoma [38]. In the present work, 800 mg/kg d-GalN caused serious hepatic damage in rats within 24 h after intraperitoneal injection. The damage was manifested by an increase of released liver enzymes, total bilirubin, marked mononuclear cellular infiltration, and congested blood vessel and bile duct hyperplasia when compared to control group. In addition, D-GalN caused increased oxidative stress, inflammation, apoptosis and necrosis of hepatocytes. This was manifested by an increased level of the lipid peroxidation marker MDA in liver tissues and by a reduced hepatic total antioxidant capacity. Furthermore, d-GalN-treated rats showed weak positive cytoplasmic immunoreaction for Bcl-2. Reduction of Bcl-2, an anti-apoptoticprotein, increases the risk of apoptotic cell death.

##### Effects on Liver Functions and Oxidative Stress Markers

**Figure 4:**
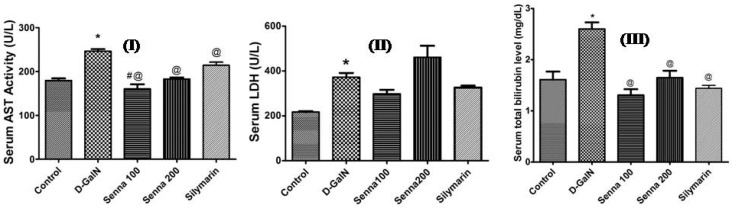
Effect of d-GalN (800 mg/kg, i.p.), bark extract (100 mg/kg and 200 mg/kg, oral), and silymarin on (**I**) AST (aspartate aminotransferase); (**II**) LDH (lactate dehydrogenase) activities (**III**) total bilirubin. Results are presented as mean ± SE. * significantly different compared to untreated control group. ^@^ Significantly different compared to d-GalN treatment group at *p* < 0.05. ^#^ Significantly different from silymarin at *p* < 0.05. *n* = 6; using One Way ANOVA followed by Tukey’s post hoc test.

Rats pre-treated with the bark extract (both concentrations 100 mg/kg and 200 mg/kg) or the positive control silymarin caused a significant reduction of AST activity and total bilirubin level when compared to d-GalN-treated group (*p* < 0.05, Figure 4). The bark extract (100 mg/kg) was more potent than silymarin in reducing AST activity (*p* < 0.05, Figure 4). A similar decline was recorded by using several crude plant extracts containing polyphenolics [25,26,39]. It was also observed that the 200 mg/kg dose level exerted a non-significant increase in LDH enzyme activity when compared to d-GalN-treated group (*p* > 0.05, Figure 4).

**Figure 5:**
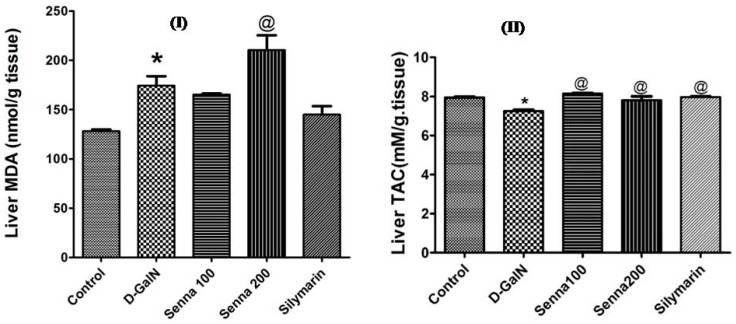
Effect of D-GalN (800 mg/kg, i.p.), bark extract (100 mg/kg and 200 mg/kg, oral), and silymarin on (**I**) MDA (malondialdehyde); (**II**) TAC (total anti-oxidant capacity) (nmol/g tissue) in the liver. Results are presented as mean ± SE. * Significantly different compared to untreated control group, ^@^ Significantly different compared to d-GalN treatment group at *p* < 0.05. *n* = 6; using One Way ANOVA followed by Tukey’s post hoc test.

Furthermore, both extract doses (100 mg/kg and 200 mg/kg) and silymarin significantly increased the total antioxidant capacity compared to theD-GalN-treated group (*p* < 0.05, Figure 5). However, the 200 mg/kg extract increased the lipid peroxidation product MDA when compared to D-GalN-treated group (*p* < 0.05, Figure 5). This result indicates that higher concentrations of the extract may have pro-oxidant activity.

##### d-GalN-Induced Histopathological Changes

**Figure 6:**
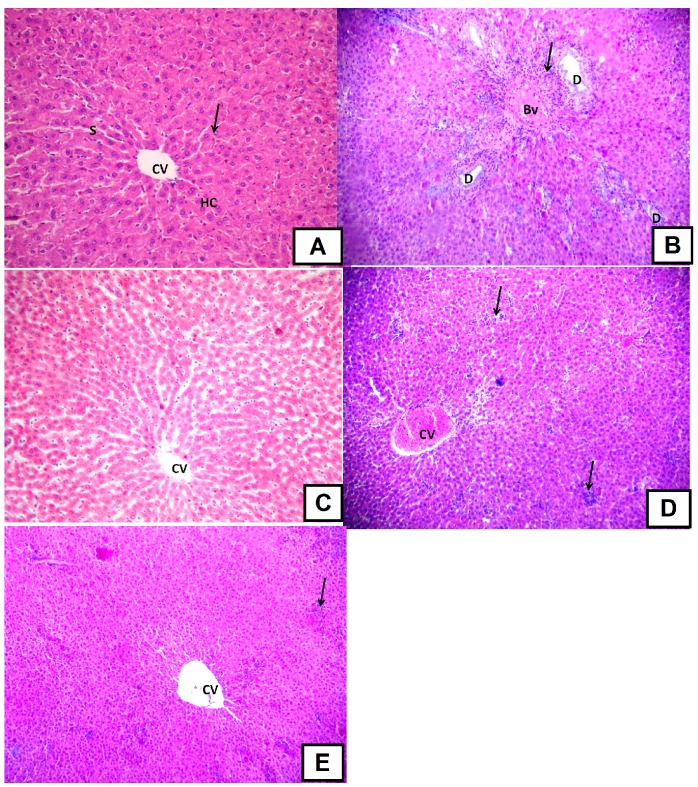
Photomicrographs of liver cross sections from 6 rats (200×). (**A**): A liver section of rat of the control group, revealing that liver tissue has normal histological structure with hepatic cords (HC) radiating from the central vein (CV) forming anastomosing plates separated by blood sinusoids (S). Hepatocytes are polyhedral with eosinophilic cytoplasm having central rounded and vesicular nuclei (arrow); (**B**): A liver section of one rat of d-GalN group, where the portal tract showed marked mononuclear cellular infiltration (arrow) and congested blood vessel (BV). Bile duct hyperplasia can be also seen (D); (**C**): Liver pre-treated with the bark extract (100 mg/kg) followed by d-GalN where the liver tissue appears nearly with normal histological structure; (**D**): Liver section of an adult rat pre-treated with *Senna singeuana* extract (200 mg/kg) followed by d-GalN group, where it showed similar pattern compared to d-GalN-treated group. There are multiple areas with mononuclear cellular infiltration (arrows) and congested central vein (CV); (**E**): Liver pre-treated with silymarin (100 mg/kg) followed by d-GalN showing partial improvement. There are some areas with mononuclear cellular infiltration (arrow).

The morphology of liver tissue of rats treated with the positive control silymarin showed only partial improvement. Also, liver of rats treated with the higher dose of the extract (200 mg/kg) did not differ from the d-GalN-treated group. There were some areas with mononuclear cellular infiltration in livers of rats treated with silymarin while multiple areas with mononuclear cellular infiltration and congested central vein were detected in livers of rats treated with the higher dose level of extract (Figure 6). Interestingly, liver of rats treated with the lower dose (100 mg/kg extract) showed the highest positive effect and was able to counteract the deleterious effects of the hepatotoxic compound d-GalN. Plant extracts rich in polyphenols and proanthocyanidins demonstrated similar activities [25,39].

##### Effects on d-GalN-Induced Apoptosis

**Figure 7:**
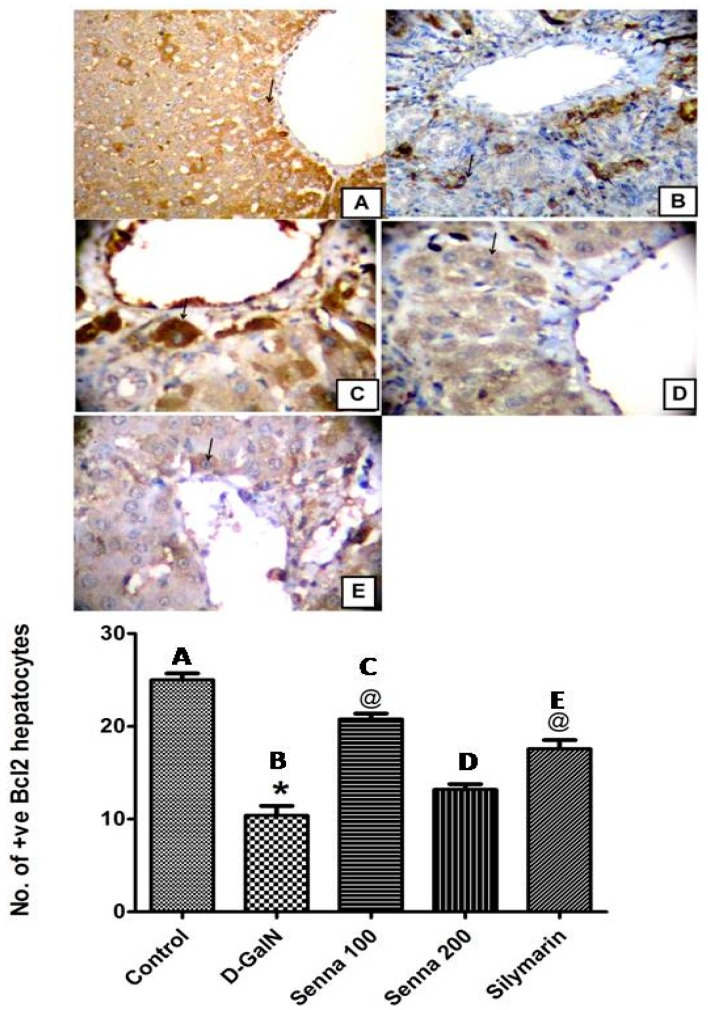
Bcl-2 expression measured by immunohistochemistry in paraffin-embedded liver tissues and stained with Avidin-biotin peroxidase stain with haematoxylin counter stain, original magnification (400×). (**A**): A liver section of an adult rat of control group showing strong positive cytoplasmic Bcl-2 immunoreaction (arrow); (**B**): A liver section of an adult rat of d-GalN group, showing weak cytoplasmic immunoreaction for Bcl-2 (arrow); (**C**): A liver section of an adult rat pre-treated with the bark extract (100 mg/kg) followed by d-GalN showing strong positive cytoplasmic immunoreaction for Bcl-2 (arrow); (**D**): A liver section of an adult rat pre-treated with the bark extract (200 mg/kg) followed by d-GalN group showing moderate cytoplasmic immunoreaction for Bcl-2 (arrow); (**E**): A liver section of an adult rat pre-treated with silymarin (100 mg/kg) followed by d-GalN showing moderate cytoplasmic immunoreaction for Bcl-2 (arrow). Five fields per group were randomly selected and analysed. Graph included with the images reveals morphometric analysis for the mean number of hepatocytes for each corresponding group which are positive to Bcl-2; statistical difference was detected using One Way ANOVA followed by Tukey’s post hoc test. * Significantly different from control group at *p* < 0.05, ^@^ Significantly different from d-GalN group at *p* < 0.05.

Bcl-2 is an essential anti-apoptotic protein that promotes survival and prevents apoptotic cell death [40]. The current study showed that increased oxidative stress associated with d-GalN administration decreased the number of hepatocytes with positive Bcl-2 expression. Reduction of Bcl-2 in liver cells by d-GalN increased apoptotic cell death. Similarly, the high dose level of the extract, which increased lipid peroxidation showed a weak stimulation of Bcl-2 similar to some extent as d-GalN. On the other hand, the low dose level of the extract (100 mg/kg) showed stronger positive enhancement of Bcl-2 than controls and silymarin (Figure 7). These results were similar to those previously reported [41,42]. To sum up, the low dose level of the extract (100 mg/kg) exerted a remarkable hepatoprotective activity against d-GalN induced hepatic injury and this might be attributed its antioxidant and antiapoptotic effects. Its effect was more potent than that of the high dose level (200 mg/kg) and silymarin.

## 3. Materials and Methods

### 3.1. Plant Material and Extraction

The bark of *Senna singueana* (Fabaceae-Caesalpinioideae) was collected from trees growing in Lupaga Site in Shinyanga, Tanzania [19]. The sample was authenticated by DNA barcoding using *rbc*L as a marker gene. A voucher specimen is kept under accession number P7328 at IPMB Heidelberg (Germany). Dried bark (100 *g*) was ground, defatted with n-hexane, and exhaustively extracted with 100% methanol (3 × 500 mL) at ambient temperature. The methanol extracts were then combined, filtered, and evaporated under vacuum at 40 °C until dryness using a rotavapor (Büchi rotavapor R-200, Büchi, Flawil, Switzerland). The residue was frozen at −70 °C, and then lyophilized yielding 10 g of a fine dried powder.

### 3.2. HPLC-PDA-MS/MS

A Thermo Finnigan (Thermo Electron Corporation, Waltham, MA, USA) LC system coupled with an LCQ-Duo ion trap mass spectrometer with an ESI source (ThermoQuest Corporation, Austin, TX, USA) was used to profile the chemical constituents of the methanol extract. A rapid resolution-reversed phase column C18 (Zorbax Eclipse XDB-C_18_, 4.6 mm × 150 mm, 3.5 µm, Agilent, Santa Clara, CA, USA) was used to separate the compounds. The mobile phase consisted of the two solvents water and acetonitrile (ACN) (Sigma-Aldrich GmbH, Darmstadt, Germany), both in 0.1% formic acid (Sigma-Aldrich GmbH, Darmstadt, Germany). A 60 min gradient from 5% to 30% ACN was used with 1 mL/min flow rate. A Surveyor autosampler (ThermoQuest) and Xcalibur^TM^ 2.0.7 software (Thermo Fischer Scientific, Waltham, MA, USA) were used to inject the sample and to control the system. The MS conditions were used as previously described [26]. Full scan mode and mass range of 50–2000 *m*/*z* were adopted to record the ions.

### 3.3. Biological Activity

#### Antioxidant Activities

In vitro antioxidant activities of the bark extract were determined using two commonly assays DPPH (2,2-diphenyl-1-picrylhydrazyl radical) and FRAP [43] following the protocol described by [44]. In brief, DPPH scavenging capacity assay was done following the standard assay reported by Blois [45] with some modifications adapted to a 96-well microplate. 100 μL DPPH solution with a concentration of 200 µM was added to the extract (100 µL) in various concentrations from 500 to 2.5 μg/mL. The samples were incubated in dark at room temperature for 30 min and the absorbance was then determined using a Safire II^TM^ spectrophotometer (Tecan, Crailsheim, Germany) at 517 nm. The following equation was used to determine the ability of a sample to scavenge the DPPH radical:

DPPH scavenging effect (%) = [(A0 − A1)/A0] × 100

The absorbance of the negative control and the extract were represented by A0 and A1, respectively. The measurements were carried out three times and EC_50_ value was calculated by sigmoid non-linear regression using adequate software.

In addition, the total phenolic content was estimated by the Folin-Ciocalteu method as described before [44]. In brief, a stock solution (2.5 mg/mL of the lyophilized extract) was prepared. Gallic acid was used as a standard compound. For extract sample and gallic acid, 20 µL was added into a 96 wells microplate and then 100 µL of Folin-Ciocalteu reagents were added. After 5 min and at the ambient temperature, 80 µL of a 7.5% sodium carbonate solution was added. The absorbance was measured after 2 h at 750 nm with a BiochromAsys UVM 340 Microplate Reader (Biowave II, Biochrom Ltd., Cambridge, UK). The results were expressed as mg gallic acid equivalent (GAE)/g extract.

In vivo antioxidant activities were evaluated using the model organism *Caenorhabditis elegans*. The nematodes were maintained under the following conditions: Worms were kept on nematode growth medium (NGM) at 20 °C, and were fed with living *E. coli* OP50 [46]. The worms were then treated with sodium hypochlorite to obtain age synchronized cultures. The eggs were kept in M9 buffer for hatching. The obtained larvae were then transferred to S-media seeded with living *E. coli* OP50 (D.O600 = 1.0) [47]. In the current work, the following strains of *C. elegans* were used: Wild type (N2), and TJ356. All of them were obtained from the *Caenorhabditis* Genetic Center (CGC), University of Minnesota, Minneapolis, MN, USA. All assays followed a protocol as reported before [44]. In brief, to determine the survival rate of the worms under juglone induced oxidative stress, we treated the worms (N2 type) with two different doses (100 and 200 µg lypholized extract/mL), except the control group and they were kept at 20 °C in S-medium. After 48 h, a toxic dose of juglone was added with a final concentration of 80 µM. After 24 h of juglone treatment, we counted the survivors and we expressed the results as percentage of live worms. Also, we investigated the intracellular ROS content in early larval stage (L1) wild type worms. The nematodes were treated with two doses of the lyophilized extract (100 and 200 µg/mL), but the control group and were incubated at 20 °C in S-medium. After 48 h, a dose of 20 µM juglone was added and after 24 h the nematodes were then incubated with 20 μM H2DCF-DA at 20 °C for 30 min in M9 buffer. Additionally, the subcellular DAF-16:GFP localization was investigated in the transgenic strain TJ356 worms. The nematodes were treated with two doses of the extract (100 and 200 µg extract/mL) at 20 °C for 24 h in S-medium. For the intracellular ROS content and DAF-16:GFP localization, fluorescence microscope (Keyence, BZ-9000, Osaka, Japan) was used to measure the fluorescence intensity and then the images were analyzed by Image J 1.48 software (National Institutes of Health, Bethesda, MD, USA) [44].

### 3.4. Hepatoprotective Activity

#### 3.4.1. *Animals*

Adult male albino rats, purchased from Faculty of Veterinary Medicine, Zagazig University, Zagazig, Egypt weighing 220–250 g were used for the present study. They were housed in polypropylene cages (5 individuals/cage) and kept under constant environmental condition of temperature (18–22 °C) and humidity (50–70%) and 12 h light/dark cycle. Rats were fed commercially available diet adlibitum and water. The animal experiments were approved by the Ethical Committee of the Faculty of Pharmacy, Zagazig University for Animal Use and conducted following the guidelines of the US National Institutes of Health on animal care and use.

#### 3.4.2. Experimental Design

Rats were randomly assigned into five groups of equal size (six rats/group) after one-week adaptation period to the environmental conditions: normal control group (1), d-galactosamine (d-GalN) group (2), *S. singueana* groups (3 and 4), and positive control group treated with silymarin, a known hepatoprotective lignin from *Silybum marianum* (5). Rats in the *Senna* groups (3 and 4) were orally gavaged with 100 and 200 mg/kg bark extract once a day for 3 consecutive days before d-GaIN was injected to induce acute hepatic failure (ALF). Group (5) received an oral dose of silymarin (100 mg/kg). The normal control and d-GalN groups received an equal amount of saline. The rats were injected (except for control) intraperitoneally with d-GalN (800 mg/kg) dissolved in normal saline.

#### 3.4.3. Blood and Tissue Sampling

At the end of the experiment, 24 h after d-GalN injection, blood was obtained from the retro-orbital plexus and centrifuged (3000× *g*, 4 °C, 20 min) to separate the serum. The collected serum was used to measure liver enzyme activities and total bilirubin. Then, rats were anesthetized with ketamine and euthanized by decapitation. Livers were dissected and put in cold saline to wash off the blood. One part of the dissected liver was kept in 10% formalin for histopathological examination and another part was immediately put in liquid nitrogen and kept at −80 °C for determination of oxidative stress markers.

#### 3.4.4. Liver Function and Oxidative Stress Markers

Liver enzymes, such as serum aspartate aminotransferase (AST) and total bilirubin concentration were measured based on the method of Young [48] and Balistreri and Shaw [49], respectively. Lactate dehydrogenase (LDH) was measured by commercial kits (Stanbio Laboratory, Boerne, TX, USA). Malondialdehyde (MDA), a lipid peroxidation product and a marker of oxidative stress, was determined according to Ohkawa et al. [50]. Total antioxidant capacity (TAC), another marker of oxidative stress, was measured by the addition of certain amount of exogenously provided hydrogen peroxide (H_2_O_2_) which reacts with the antioxidants in the serum sample. A part of the provided hydrogen peroxide was eliminated by the antioxidants of the sample and the remaining amount of H_2_O_2_ was measured by colorimetric method that involves an enzymatic reaction which converts 3,5-dichloro-2-hydroxybenzenesulphonate to a coloured compound [51].

#### 3.4.5. Histological Techniques

The isolated liver tissues were fixed using 10% neutral formaldehyde. Then, the tissues were passed in ascending grades of ethyl alcohol (70%, 90%, and 100%) for dehydration. Then, they were cleared in xylol, soaked and then fixed in paraffin wax. 5-micron pieces were sectioned using a rotary microtome. The sections were stained with H&E stain to study the general microscopical structure of hepatic tissues and then examined under light microscope [52].

#### 3.4.6. Immunohistochemical Techniques

In order to determine the expression of Bcl-2, an oncoprotein that inhibits the programmed cell death (apoptosis) [53], the liver sections were deparaffinized and rehydrated. Then, 5% H_2_O_2_ in absolute methanol was added for 10 min at room temperature to block the activity of endogenous peroxidase activity. Phosphate buffered saline (PBS) was added to rinse the sections. Thereafter, they were incubated with primary antibodies against Bcl-2. A streptavidin biotin peroxidase kit was used to measure protein expression level. Diaminobenzidine (DAB) as chromogen for Bcl-2 detection was used to stain the tissues and then counterstained with hematoxylin [54].

#### 3.4.7. Morphometric Study

A computerized image system composed of a Leica Qwin 500 image analyser connected to a Leica microscope (Leica, Cambridge, UK) was used to detect the number of Bcl-2 positive hepatocytes in five randomly selected high power microscopic fields within the liver sections. This number was expressed as cell number per µm² [55].

### 3.5. Statistical Analysis

Data are presented as mean ± standard error of mean. GraphPad Prism statistical program software (version 5, GraphPad Software, Inc., San Diego, CA, USA,) was used to determine statistical difference between various groups. One-way analysis of variance test (ANOVA) was used to detect statistical significance of multiple groups followed by Tukey’s Post Hoc Test to determine statistical difference between each 2 individual groups. The Probability level less than 0.05 was accepted as a level of significance.

## 4. Conclusions

The chemical constituents of the bark extract of *Senna singueana* were investigated using HPLC-MS/MS. The bark extract is rich in proanthocyanidins. The extract showed promising antioxidant activities in vitro and in *C. elegans.* Furthermore, remarkable hepatoprotective and antiapoptotic activities were detected. Thus, the bark extract and probably the tannins are good candidates to explore diseases in more detail associated with liver injury and oxidative stress.

## Figures and Tables

**Table 1:** Identification of secondary metabolites of the methanol extract from the bark of *Senna singueana* by HPLC-MS/MS.

No.	R_t_ (min)	[M − H]^−^	MS/MS	Proposed Compounds	Ref.
** 1**	1.33	133	115	Malic acid	[26]
** 2**	1.68	447	163, 315	*p*-Coumaric acid galloyl-pentoside	
** 3**	2.48	153		Protocatechuic acid	
** 4**	4.52	305	179, 221, 287	(epi)Gallocatechin	[25]
** 5**	5.33	417	161, 205, 270	Unknown	
** 6**	5.90	593	289, 441	(epi)Catechin digallate	
** 7**	8.18	305	125, 179, 287	(epi)Gallocatechin	[25]
** 8**	8.48	593	305, 425, 575	(epi)Gallocatechin-(epi)catechin	[25]
** 9**	9.57	289	179, 205, 245	(epi)Catechin	[26]
** 10**	11.52	577	289, 425, 559	(epi)Catechin-(epi)catechin	[26]
** 11**	13.10	577	289, 425, 559	(epi)Catechin-(epi)catechin	[26]
** 12**	14.65	289	179, 205, 245	Catechin	[26]
** 13**	15.76	561	179, 305, 543	(epi)Guibourtinidol-(epi)gallocatechin	
** 14**	17.14	561	289, 451, 543	(epi)Catechin-(epi)afzelechin	[26]
** 15**	17.90	561	179, 305, 543	(epi)Guibourtinidol-(epi)gallocatechin	
** 16**	20.16	561	179, 305, 543	(epi)Guibourtinidol-(epi)gallocatechin	
** 17**	20.87	545	289, 409, 527	(epi)Guibourtinidol-(epi)catechin	[27]
** 18**	21.82	561	179, 305, 543	(epi)Guibourtinidol-(epi)gallocatechin	
** 19**	24.20	545	179, 289, 527	(epi)Guibourtinidol-(epi)catechin	[27]
** 20**	26.37	545	179, 289, 527	(epi)Guibourtinidol-(epi)catechin	[27]
** 21**	27.82	545	179, 289, 527	(epi)Guibourtinidol-(epi)catechin	[27]
** 22**	28.64	545	179, 305, 527	(ent)Cassiaflavan-(epi)gallocatechin	[28]
** 23**	29.30	545	179, 305, 527	(ent)Cassiaflavan-(epi)gallocatechin	[28]
** 24**	31.31	833	305, 561, 577	(epi)Gallocatechin-(epi)catechin-(ent)cassiaflavan	
** 25**	33.96	529	289, 419, 511	(ent)Cassiaflavan-(epi)catechin	[28]
** 26**	34.72	817	305, 561, 663	(epi)Guibourtinidol-(epi)guibourtinidol-(epi)gallocatechin	
** 27**	37.58	833	305, 593, 723	(epi)Gallocatechin-(epi)catechin-(ent)cassiaflavan	
** 28**	37.73	529	179, 289, 511	(ent)Cassiaflavan-(epi)catechin	[28]
** 29**	40.56	801	289, 545, 691	(epi)Guibourtinidol-(epi)guibourtinidol-(epi)catechin	
** 30**	45.35	801	305, 561, 691	(ent)Cassiaflavan-(epi)guibourtinidol-(epi)gallocatechin	
** 31**	46.41	785	305, 545, 767	(ent)Cassiaflavan-(ent)cassiaflavan-(epi)gallocatechin	
** 32**	48.75	801	305, 425, 561	(ent)Cassiaflavan-(epi)guibourtinidol-(epi)gallocatechin	
** 33**	53.87	785	289, 545, 675	(ent)Cassiaflavan-(epi)guibourtinidol-(epi)catechin	
** 34**	55.50	785	305, 545, 675	(ent)Cassiaflavan-(ent)cassiaflavan-(epi)gallocatechin	
** 35**	56.50	785	305, 545, 675	(ent)Cassiaflavan-(ent)cassiaflavan-(epi)gallocatechin	
**36**	58.62	769	289, 529	(ent)Cassiaflavan-(ent)cassiaflavan-(epi)catechin	

Prefixes (epi) and (ent) indicate that the stereochemistry of the compounds is not resolved.
